# Phage Reduce Stability for Regaining Infectivity during Antagonistic Coevolution with Host Bacterium

**DOI:** 10.3390/v11020118

**Published:** 2019-01-29

**Authors:** Yihui Yuan, Qin Peng, Shaowen Zhang, Tingting Liu, Shuo Yang, Qiuhan Yu, Yan Wu, Meiying Gao

**Affiliations:** 1 State Key Laboratory of Marine Resource Utilization in South China Sea, Hainan University, Haikou 570228, China; yuanyh@hainu.edu.cn (Y.Y.); xiaoying_zhang@163.com (S.Z.); LttLc2018@163.com (T.L.); yangshuohainu@126.com (S.Y.); yuqiuhan0914@foxmail.com (Q.Y.); 2 Wuhan Institute of Virology, Chinese Academy of Sciences, Wuhan 430071, China; wuyan_81@126.com; 3 Ministry of Education Key Laboratory for Ecology of Tropical Islands, College of Life Sciences, Hainan Normal University, Haikou 571158, China; pengqin1019@126.com

**Keywords:** bacteriophage, coevolution, synergism mutation, baseplate, flagellum

## Abstract

The coevolution between phage and host bacterium is an important force that drives the evolution of the microbial community, yet the coevolution mechanisms have still not been well analyzed. Here, by analyzing the interaction between a *Bacillus* phage vB_BthS_BMBphi and its host bacterium, the coevolution mechanisms of the first-generation phage-resistant bacterial mutants and regained-infectivity phage mutants were studied. The phage-resistant bacterial mutants showed several conserved mutations as a potential reason for acquiring phage resistance, including the mutation in flagellum synthesis protein FlhA and cell wall polysaccharide synthesis protein DltC. All the phage-resistant bacterial mutants showed a deleted first transmembrane domain of the flagellum synthesis protein FlhA. Meanwhile, the regain-infectivity phage mutants all contained mutations in three baseplate-associated phage tail proteins by one nucleotide, respectively. A polymorphism analysis of the three mutant nucleotides in the wild-type phage revealed that the mutations existed before the interaction of the phage and the bacterium, while the wild-type phage could not infect the phage-resistant bacterial mutants, which might be because the synchronized mutations of the three nucleotides were essential for regaining infectivity. This study for the first time revealed that the synergism mutation of three phage baseplate-associated proteins were essential for the phages’ regained infectivity. Although the phage mutants regained infectivity, their storage stability was decreased and the infectivity against the phage-resistant bacterial mutants was reduced, suggesting the phage realized the continuation of the species by way of “dying to survive”.

## 1. Introduction

Bacteriophages, the parasites of bacteria, are the most abundant and diverse biological entity in the natural ecosystem [1]. The same as the relationships of the other parasites and hosts, the phages and their host bacteria realize coexistence by antagonistic coevolution, which is defined as the reciprocal evolution of host resistance and parasite infectivity [2,3,4,5]. The coevolution between phage and bacterium is one of the most important forces that drives the evolution of the microbial community and is also considered as one crucial factor that causes and maintains the biodiversity of microorganisms [2]. In addition to the role of providing an understanding of the ecology and evolution of microbial populations, study of phage–bacterium coevolution could also increase our knowledge on the role of phages in the evolution of bacterial virulence and the use of phages for clinical purpose [6,7]. For the purpose of understanding the reasons and consequences of phage–bacterium coevolution, the coevolution between bacteria and their lytic viral parasites have been extensively studied on several phage–host interaction models [4,8,9]. The coevolution with phages may cause many important phenotypic changes to the host bacteria, including phage resistance, bacterial social behavior, diversity and niche competition, and the mutation rates in evolution [10,11,12]. However, the coevolution of the phage and bacterium have only been analyzed for limited bacterial species, which could not reflect the diversity of phage–bacteria coevolution. Besides this, previous study on phage–host coevolution mainly focused on the dynamic and population changes of the phages and their hosts, and few detailed analyses on the genomic changes of the phage and the host bacterium have been studied.

As a virus that infects and lyses a pathogenic bacterium, the phage is considered as a novel kind of antimicrobial substance which conquers the appearance of bacterial antibiotic resistance [13,14]. However, because of the coevolution of a phage and their host bacterium, the resistance of the pathogenic bacteria to phages appeared at a high frequency [15]. Although several approaches have been applied to deal with the appearance of phage resistance during the clinical use of phage preparations, such as the use of a phage cocktail that contains different types of phages, these strategies could not fundamentally solve the problem. Thus, for the purpose of developing efficient phage therapy, it is urgent to analyze the coevolution mechanism of phages and their host bacteria.

*Bacillus thuringiensis* is the pathogenic bacterium of insects and is widely used in producing biological pesticides [16]. *B. thuringiensis* is closely related with the human pathogenic bacteria *Bacillus anthracis* and *Bacillus cereus* in evolution, and these three species accompany *B. weihenstaphanensis*, *B. pseudomycoides*, and *B. cytotoxicus* in being classified as the *Bacillus cereus* group [17]. *B. anthracis* is the pathogen of human anthrax, and *B. cereus* can cause the emesis and diarrhea of humans by the production of emetic toxin and enterotoxins [18,19]. Previous study on phages infecting *B. thuringiensis* revealed that some of the phages could also infect the strains from *B. anthracis* and *B. cereus* [20]. Furthermore, although numerous phages infecting *Bacillus* species strains have been isolated, no study on the coevolution between *Bacillus* strains and their phages have been reported.

In this study, the coevolution between the phage vB_BthS_BMBphi and the host bacterial strain *B. thuringiensis* BMB171 was analyzed. The phage-resistant bacteria and the regained-infectivity phage mutants appeared at a high frequency during the co-cultivation of the phage and the host bacterium. Both the characteristics and the genome of the phage-resistant mutants and the regain infectivity phage mutants were analyzed. The result shows that the phage-resistant bacterial mutants present diverse mutations, while the regained-infectivity phages mutants exhibit conserved mutants. It is interesting to note that the regained-infectivity phage mutants show reduced stability by the mutation of the tail associated proteins, which is responsible for the ability to regain infectivity. The finding of this study not only revealed the coevolution mechanism of the phage and the bacterium, but also figures out a new kind of “dying to survive” strategy for maintaining race continuity.

## 2. Materials and Methods

### 2.1. Bacterial Strains and Growth Conditions

The *B. thuringiensis* strain BMB171 is a widely used acrystalliferous mutant for the construction of genetic engineering strains to express insecticidal crystal protein [21]. The phage vB_BthS_BMBphi is a *Siphoviridae* family phage with a genome size of 49,277 bp (GenBank accession number MH458951) and was isolated by our lab [22]. The strains were cultivated in Luria–Bertani (LB) broth medium at 30 °C with moderate shaking at 180 rpm. The agar with a final concentration of 1.2% and 0.7% were added into the LB broth to prepare the solid and semisolid medium, respectively. The agar at a final concentration of 0.3% was added into the LB broth for the preparation of the semisolid medium for testing the mobility of the bacterial strains. The changes in the mobility of the bacteria were analyzed with SPSS by using ANOVA (Version 19.0, Chicago, IL, USA).

### 2.2. Phage Propagation and Concentration Determination

The phage propagation and efficiency-of-plating (EOP) test were all performed by using the double-layer overlay method [23]. The storage stability of the phage was also determined by testing the EOP of the phage suspension after storage for different time periods.

### 2.3. Screening of Phage-Resistant Bacterial Mutants and Regained-Infectivity Phage Mutants

For the purpose of screening bacterial mutants that are resistant to phages, a single clone of BMB171 formed on agar plate was inoculated into LB broth and cultivated at 30 °C with moderate shaking at 180 rpm. After cultivation for 12 h, the exponential growth strain BMB171 with a concentration of 5 × 10^8^ CFU/mL was mixed with the phage vB_BthS_BMBphi at the multiplicity of infection (MOI) of 1000 and left at 30 °C for 30 min for the adsorption and infection of the phage to the strain. The mixture was then mixed with the melted semisolid agar medium at about 45 °C and poured onto the solid agar plate. After growth at 30 °C overnight, the bacterial clones which formed on the semisolid plate were picked and inoculated onto a semisolid plate that contained phage vB_BthS_BMBphi with a concentration of 10^8^ PFU/mL. By repeating the cultivation process on phage-containing plates more than 5 times, the bacterial isolates that could form well growth clones on the agar plate were thought to be resistant to the phage vB_BthS_BMBphi and named as phage-resistant bacteria (PRB).

The phage mutants that regained infectivity to the phage-resistant bacterial mutants were isolated by the co-cultivation of the phage and the phage-resistant bacterial mutants. Generally speaking, the phage was mixed with the exponential-growth phage-resistant strain (5 × 10^8^ CFU/mL) at the MOI of 1.0 and added into the LB broth as a ratio of 1%, and subsequently cultivated at 30 °C with moderate shaking at 180 rpm for 24 h. The co-cultivation process was only performed once and no replicate was carried out. The co-cultivation process could enrich the phage that could infect the phage-resistant mutants. The cultures were collected by centrifugation at 12,000× *g* for 10 min and filtered through a 0.22-μm filter. The infectivity of the phage in the filtered supernatant was tested by using the phage-resistant bacterial mutants as indicator strains, and the plaques which formed on the bacterial lawn were picked and purified more than 5 times by the double agar overlay method by using the phage-resistant bacterial mutants as indicator strains [23]. The infectivity of the phage to the PRB strains derived from BMB171 were retested three times, and phages continuously infecting the PRB strains were thought of as regained-infectivity phage mutants of phage vB_BthS_BMBphi.

### 2.4. Binding Ability Assay of the Phage to Bacteria

The binding ability of the phage to the bacterial strains were tested by testing the EOP of the phage suspension after the addition of the bacterial cultures. The exponential growth strain with a concentration of 5 × 10^8^ CFU/mL was mixed with an equal volume of phage suspension with a concentration of 5 × 10^7^ PFU/mL at 30 °C. The sample was collected at an interval of 5 min and then centrifuged at 12,000× *g* for 1 min to remove the cells in the mixture, and the phage titers of the supernatant were determined immediately. The experiment was carried out in triplicate.

### 2.5. Bacterial and Phage Genomic DNA Purification, Genome Sequencing, and Bioinformatic Analysis

The genomic DNA of the phages and the host bacteria were purified as per the methods previously described [23,24]. The genomes of the bacteria and phages were sequenced by using Illumina Hiseq 2500 (Illumina, San Diego, CA, USA) and assembled into contigs using software Velvet v1.2.07 [25]. The visualization of the phage genome was performed by using CGview [26]. The mutant sites of the phage-resistant bacteria and regained-infectivity phages were analyzed by mapping the reads archived by genome sequencing onto the genome of the phages and bacteria using Geneious version R11 [27].

## 3. Results

### 3.1. Phage-Resistant Bacterial Mutants and Regained-Infectivity Phage Mutants Appeared at High Frequency

**Figure 1:**
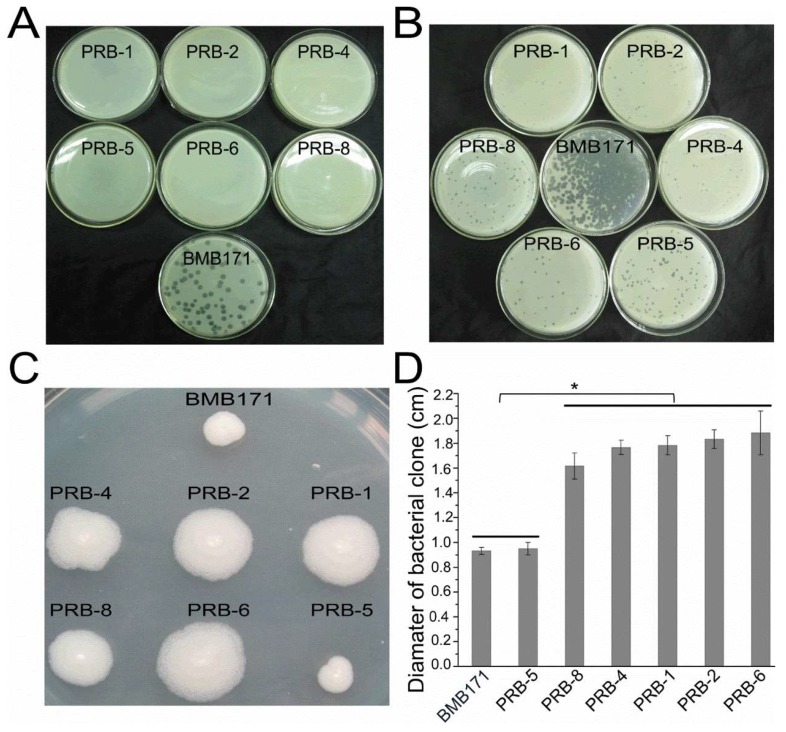
Isolation and characterization of phage-resistant mutants of BMB171 and regained-infectivity phage mutants of vB_BthS_BMBphi. (**A**) Phage-resistant mutants of BMB171 isolated in this study. (**B**) Infectivity of regained-infectivity phage mutants vB_BthS_BMBphi-M1 to the phage-resistant strains and BMB171. (**C**) The mobility of the strains BMB171 and the phage-resistant mutants. The agar plate with a concentration of 0.3% was used for bacterial mobility analysis. (**D**) Comparison of the bacterial clone size of strain BMB171 and the phage-resistant mutants. For each strain, 30 clones were used for the analysis of the clone diameters, and the asterisk indicates a significant difference (*p* < 0.05) between the diameter of different bacterial clones.

Phage vB_BthS-BMBphi is a *Siphoviridae* family phage that specifically infects the *B. thuringiensis* strain BMB171. The phage exhibits high lytic activity to the strain BMB171 and contains a novel genome sequence, which only shows low similarity with two *B. thuringiensis* prophages. During the co-cultivation of the phage vB_BthS_BMBphi and host strain BMB171, numerous clones were found on the plate that contained a high concentration of phage vB_BthS_BMBphi (higher than 10^8^ PFU/mL). The ratio of the phage-resistant clones which formed on the plate was about 1.7 × 10^−8^ of the original added strain numbers. However, when the cultures of BMB171 with a concentration of 5 × 10^9^ CFU/mL were spread onto the plate containing the phage vB_BthS_BMBphi with a concentration of 10^8^ PFU/mL, no phage-resistant clones formed on the plate, which is not consistent with the previously determined ratio of phage-resistant clones (1.7 × 10^−8^). We supposed that during the cultivation of the phage and bacterium on the plate, the endolysin produced by the phage infecting sensitive bacterial strains caused the lysis of phage-resistant strains [28]. The lytic activity assay of the endolysin PlyBMB encoded by phage vB_BthS_BMBphi against the phage-resistant strain also confirmed that the phage-resistant bacterial mutants could be lysed by the endolysin (Figure S1). The clones were picked and the resistance of the picked clones to the phage vB_BthS_BMBphi was confirmed, and the result showed that only six of the nine picked clones showed persistent resistance to the phage (Figure 1A), while the other picked strains were pseudo-resistant strains and could be infected by a high concentration of phage vB_BthS_BMBphi. The six phage-resistant bacterial mutants (named as PRB-1, PRB-2, PRB-4, PRB-5, PRB-6, and PRB-8) that exhibited stable resistance to the phage were used for the further isolation of regained-infectivity phage mutants.

After the co-cultivation of the phage vB_BthS_BMBphi and phage-resistant strain PRB-4 for 24 h, the suspension of the culture was used to screen regained-infectivity phage mutants. Initially, 100 plaques formed on the bacterial lawn of strain PRB-4 were picked, and their infectivity to all six bacterial mutants of BMB171 were analyzed. The results showed that all the 100 picked phage isolates could infect all the six phage-resistant mutants, (Figure 1B). In the following study, four regained-infectivity phage mutants (named as vB_BthS_BMBphi-M1 to vB_BthS_BMBphi-M4) were randomly picked and used for the following study.

### 3.2. Phage-Resistant Bacterial Mutants Exhibiting Changed Features

**Figure 2:**
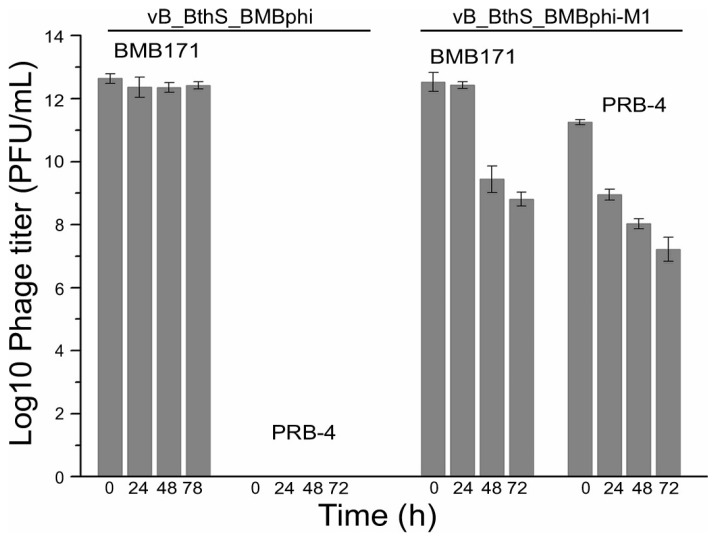
Storage stability of the phage vB_BthS_BMBphi and vB_BthS_BMBphi-M1. The infectivity of the two phages was tested against the strains BMB171 and PRB-4, respectively, after storage for 24, 48, and 72 h.

According to previous reports, phage-resistant mutants might generate some changes for the bacterial phenotype, including mobility [29]. In this study, the mobilities of the phage-resistant mutants were analyzed, and the result showed that the mutants exhibited different mobilities on the semisolid medium plate. Strains BMB171 and PRB-5 formed small clones on the plate, while the other five mutants showed significantly bigger clones (Figure 1C,D). The diameter of the bacterial clone also indicates that the bacteria might have an improved replication rate. The infectivity of the wildtype phage and phage mutants to the strain BMB171 and strain PRB-4 were also analyzed. Phage vB_BthS_BMBphi could only infect the wild type strain BMB171, but not the phage-resistant mutant strains PRB-1. The result also showed that the mutant phage vB_BthS-BMBphi-M1 showed higher infection ability against the wild-type strain BMB171 than to the mutant strain PRB-4. The infectivity of phage vB_BthS_BMBphi-M1 to the strain PRB-4 was only 4.67% of that of strain BMB171 (Figure 2). The storage stability analysis of phage vB_BthS_BMBphi and vB_BthS_BMBphi-M1 revealed that the wild-type phage was highly stable, while the mutant phage was unstable and the phage titer decreased sharply during storage. After storage for 72 h, only 5.2 × 10^−5^ of the phage remained infective compared to the initial phage suspension.

**Figure 3:**
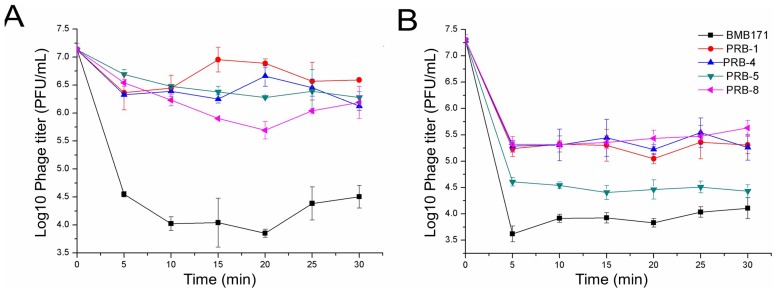
Absorption of phage vB_BthS_BMBphi (**A**) and vB_BthS_BMBphi-M1 (**B**) to strain BMB171 and four phage-resistant mutants.

The loss of the binding ability of phages to the host bacteria is one of the most common reasons for the phage resistance [10]. The binding ability of the phages to the strain BMB171 and four phage-resistant mutants has been analyzed. The wild-type phage vB_BthS_BMBphi only showed a binding ability to the wild-type strain BMB171 (Figure 3A), while the mutant phage vB_BthS_BMBphi-M1 showed a binding ability to all the tested strains (Figure 3B), suggesting the changes of binding ability might be the reason for the phage resistance and the reason for regaining infection ability. Although the mutant phage exhibited binding ability to all the tested strains, they showed different levels of binding ability. The phage vB_BthS_BMBphi-M1 showed the highest binding ability to the strain BMB171, and subsequently PRB-5, and then the other three mutant strains. Compared with the other three mutant strains, strains PRB-5 and BMB171 showed lower mobility, suggesting that the increase of mobility might lead to the decrease of the phage-binding ability, and subsequently the decrease of infectivity.

### 3.3. Phage-Resistant Bacterial Mutants Possessing Diverse Mutation Sites

The genomes of the phage-resistant bacteria were individually sequenced to determine the mutation sites that caused the phage resistance (Table S1). The result showed that although the phage-resistant mutants exhibited diverse mutation sites, several of the mutations located in the functional genes were conserved. In total, 183 nucleotides, including 117 nucleotides located in the non-coding regions, 14 synonymous mutations, and 52 non-synonymous mutations, were found to be mutated by single nucleotide polymorphism (SNP) analysis (Table S2). The binding-ability assay of the phage vB_BthS_BMBphi and the regained-infectivity phage mutants to the phage-resistant bacterial mutants revealed that the loss of binding ability leads to the loss of infectivity (Figure 3), suggesting the phage-resistant bacterial mutant might be generated by the mutation of the phage-binding receptor on the bacterial cell surface. In consideration of the resistance of all these four genome-sequenced bacterial mutants, the conserved mutations in these bacterial mutants might determine the phage resistance. Among the 52 non-synonymous mutations, 34 of them are conserved in four of the phage-resistant bacterial mutants, and only part of these mutations were located in cell wall-associated protein, including cell wall anchor (BMB171_C0382), arsenic transporter (BMB171_C2830), bacitracin ABC transporter permease (BMB171_C3335), cell surface protein (BMB171_C4543), and multidrug MFS transporter (BMB171_C4873). However, none of these proteins were reported to be associated with the infection of the phages. The bacterial cell wall polysaccharide was thought to be the binding receptor of a large number of phages [30]. Mutation analysis showed that the gene BMB171_C1214, which was annotated as d-alanine–d-alanyl carrier protein (DltC) and was responsible for the synthesis of the cell wall polysaccharides [31], was mutated in all of the four genome-sequenced phage-resistant bacterial mutants (supplementary Table S1, Supplementary materials). The mutation in PRB-1, PRB-4 and PRB-5 was the deletion of a guanine thymine nucleotide (T), which caused the truncation of the protein DltC. However, in strain PRB-5, the mutation was the change from guanine nucleotide (G), which was non-synonymous and caused the change of residue of Threonine (Thr) to Valine (Val). Thus, the mutation of DltC might be one potential reason of the archiving of phage resistance.

**Figure 4:**
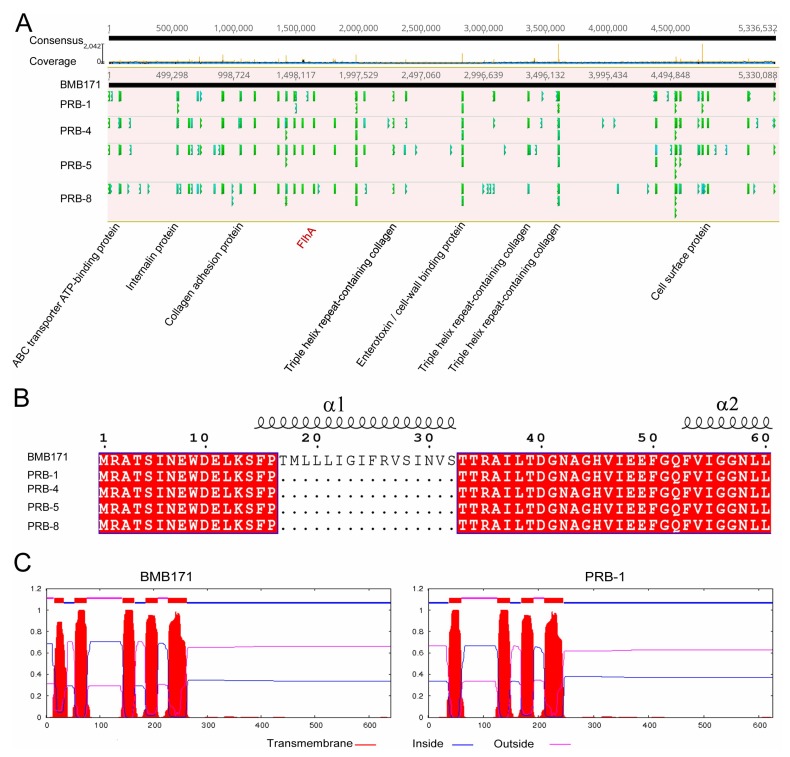
Analysis of the mutations of the phage-resistant bacterial mutants. (**A**) The mutant sites in four phage-resistant bacterial mutants. The functions and locations of the cell surface proteins that mutated in all the four mutants are indicated. (**B**) Alignment of the protein FlhA from the strains BMB171 and four phage-resistant mutants. The secondary structures of the protein are indicated. (**C**) Transmembrane domain of protein FlhA from strain BMB171 and PRB-1.

By mapping the reads archived by the genome sequencing of these four strains onto the genome of strain BMB171, several new mutations were found (Figure 4A). A comprehensive analysis of the mutations found by genome mapping revealed that the first transmembrane α-helix domain at the N-terminal of the bacterial flagellar biosynthesis protein FlhA (BMB171_C1535) was deleted in all the four phage-resistant bacterial mutants (Figure 4B,C). FlhA is required for the synthesis of flagellum by exporting flagellum synthesis associated proteins [32]. Flagellum was proven to be the phage-binding receptor of some phages, and the deletion of FlhA was found to lead to the resistance of the bacterium to phages [33]. In consideration of the change of phage-binding ability, it is rational to deduce that the mutation of FlhA might be the reason that caused the phage resistance of BMB171. Although FlhA is reported to be associated with the mobility of the bacterium, three of the four bacterial mutants showed possibly increased mobility and one showed possibly decreased mobility. It was possible that the changes in the mobility of the bacterial mutants were associated with the mutation of the other genes, which needs to be further analyzed.

### 3.4. Regained-Infectivity Phage Mutants Showing Conserved Mutation Sites

**Figure 5:**
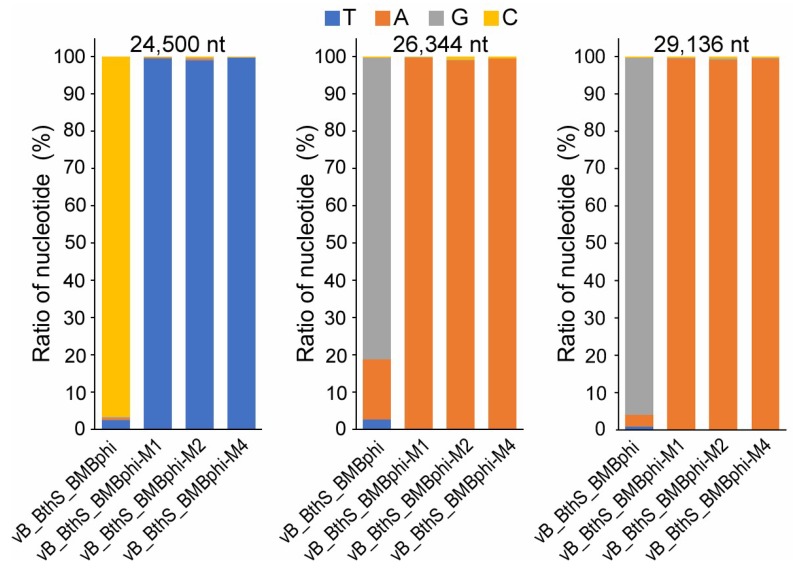
Polymorphism of the mutant nucleotides in phage genome. The ratio of the nucleotide compositions of the three mutant nucleotides in four phage genomes is shown. The nucleotide composition was obtained by analyzing the raw reads archived by genome sequencing and the sites of each mutant nucleotides were shown.

To figure out the mechanism of regaining infectivity, the genomes of all the four picked phage mutants were sequenced (Table S3). Genomic analysis of the four mutant phage isolates revealed that they contained conserved mutation sites, including the mutations of one cytidylate nucleotide (C in short) at the 24,551th nucleotide of the genome to thymidine nucleotide (T in short) and two guanine nucleotides (G in short) at 26,344 and 29,136 residues to adenine nucleotides (A in short) in the mutant phage isolates (Figure 5). The residue 24,551 was located in the phage baseplate protein (Gp44), which was the first phage structure protein in the phage vB_BthS_BMBphi structural gene module. The other two mutational sites were located in the tail protein (Gp46) and the distal tail protein (Gp47). The raw reads generated by high-throughput genome sequencing were mapped onto the mutational nucleotides of the phage genome. The result showed that the nucleotides site 24,551 exhibited little polymorphism and only few reads (1.48% of all the reads) showed different nucleotide compositions as the phage vB_BthS_BMBphi genome. However, the sites of 26,344 and 29,136, which were both G in the assembled wild-type phage genome, contained a high ratio of A, and were the nucleotides at the genome of the mutant phage. For the sites 24,551, 26,344, and 29,136 in the phage vB_BthS_BMBphi genome, 2.47%, 16.07%, and 3.05% of the mapped nucleotides were the same as the nucleotides in the mutant phage genome, suggesting the individual mutant existed before the screening of the phage by using a phage-resistant bacterial mutant. Based on the ratio of each nucleotide site, the frequency of the co-existence of these three mutations was 1.21 × 10^−4^, indicating that 0.0121% of the phage virions of phage vB_BthS_BMBphi might infect the phage-resistant bacterial mutants. The infectivity activity of the phage vB_BthS_BMBphi to the phage-resistant strains PRB-4 was also determined. By spreading 5 × 10^11^ PFU phage vB_BthS_BMBphi onto the plate containing 5 × 10^8^ CFU strain PRB-4, no phage plaque was observed on the plate. The result suggests that the existing of regained-infectivity phage before the screening process is lower than a frequency of 2 × 10^−12^, which was significantly lower than the theoretical frequency of 1.21 × 10^−4^, suggesting there was no regained infectivity phage before co-cultivation process or the regained-infectivity phage was propagated during the co-cultivation process.

**Figure 6:**
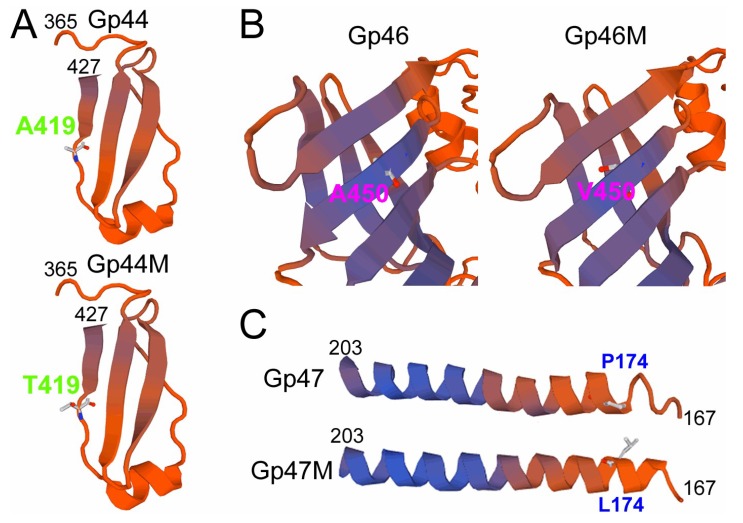
Predicted structure of the regions in the mutant proteins from the phage genome. Structural modelling of the baseplate protein Gp44 (**A**), tail endopeptidase protein Gp46 (**B**) and distal tail protein Gp47 (**C**). The structures of the peptides containing the mutant sites are shown. The positions of the mutant amino acid residues and the positions of the shown peptides in the proteins are indicated.

Function analysis of the three proteins showed that all of them were associated with the formation of the phage baseplate. Protein Gp44 was annotated as the phage baseplate protein and was reported to be the receptor-binding protein in several of the other phages [30,34]. The mutation of Gp44 happened at residue 419 by the mutation of the hydrophobic amino acid Alanine (Ala) to hydrophilic amino acid Thr (Figure 6A). The mutation of A419T was located near the terminus of the protein. The change of the amino acid might influence the hydrophilicity of the protein and subsequently the interaction of the protein with the other proteins. The gene *gp*46 was located between the baseplate protein-encoding gene *gp*44 and distal tail protein-encoding gene *gp*47 in the phage genome. Bioinformatic analysis of Gp46 revealed that the protein was homologous with the phage tail endopeptidase (PDB database entry 3GS9), which is required for the efficient infection of the phage and also associated with the formation of the baseplate [35]. The mutation in the tail protein Gp46 caused the change of residue 450 from Ala to (Valine) Val. Structural modelling of the wild-type protein revealed that the mutant residue was located in a β-sheet at the surface of the protein (Figure 6B). The protein Gp47 was annotated as a phage distal tail protein, which formed the hub of the baseplate and contained carbohydrate binding modules for phage infection [36,37]. The mutation in Gp47 was the change of Proline (Pro) to Leucine (Leu) at residue 174, which was located in the α-helix region at the C-terminus of the protein and near the linker region between the N-terminus and C-terminus of the protein. The N-terminus of the distal tail protein formed a cylinder channel for DNA translocation during the infection [37]. The C-terminus was located outside the channel to form a hub protrusion that might contain a carbohydrate-binding module [38]. The mutant residue P174L might change the secondary structure of the protein and formed a prolonged α-helix, which might influence the conformation of the protein (Figure 6C).

## 4. Discussion

As the most diverse biological entities in the Earth’s ecosystem, the coevolution between phages and bacteria exhibits high diversity [39,40]. In this study, the coevolution between the phage vB_BthS_BMBphi and its host strain BMB171 was analyzed. An analysis of the variations in mutant bacteria and mutant phages revealed that the bacterial strains contained more mutations in different nucleotides sites and different forms of mutation. Except for the mutation associated with phage resistance, the mutant bacteria also exhibited diverse changes in other phenotypes, such as the change of bacterial mobility. Several transporters which were associated with the transport of chemical substances were also mutated. The CRISPR system is one of the most important mechanisms that endows the bacteria with resistance to the phages [41]. The genomic analysis of the phage-resistant bacterial mutants by analyzing the repeats in the bacterial genome showed that there are no repeats from the phages found in the genome of the bacterial mutants, suggesting the CRISPR is not responsible for the archiving of resistance. However, the phages showed conserved mutations in a few nucleotides, and all the mutations in the regained-infectivity phages analyzed in this study were the change of a single amino acid residue. Compared to phages, the bacteria contained more copies of homologous genes with complementary functions, while the phages contained a minimalistic genome structure, and genes with similar functions only existed in a single copy. The mutation of the critical residue in the phage protein very possibly caused the lethal mutation of the phage [42], which caused the phage to realize coevolution by minor mutation.

The change in the phage-binding receptor on the bacterial cell surface is one of the most common reasons that caused the resistance of bacteria [5,28,43,44]. Several studies revealed that the absorption of flagellum is the first step for phage infection, and the deletion of flagellum would lead to the generation of phage resistance [29,45]. However, there is no study on what kind of mutation in flagellum will cause the phage-resistance of a bacterium. The finding of this study revealed that the deletion of the first transmembrane domain of flagellum synthesis protein FlhA might cause the generation of phage resistance. FlhA is a component of the flagellar protein-export apparatus, with an integral membrane domain encompassing the N-terminal transmembrane domain (FlhA_TM_) and a cytoplasmic C-terminal domain (FlhA_C_) [32]. Only a few studies had reported that FlhA_TM_ could interact with the other proteins in the flagella protein export apparatus to form the MS ring across the bacterial cell membrane for exporting flagellar protein [46], and the detailed function of the transmembrane domain in FlhA had not been analyzed. The mutations of the residues between the transmembrane segments could cause the global change of the FlhA_C_ domain, which interacts with the other proteins of the flagellar export apparatus and influences the formation of the flagellar export apparatus [47]. The transmembrane domain of FlhA was located in the center of the flagella protein export apparatus and is unlikely to be contracted by the phage directly [32]. We speculated that the deletion of the first transmembrane domain of FlhA led to the conformational change of the protein and, subsequently, the change of the flagellum, which led to the changes of phage sensitivity.

The phage tail proteins were proven to take part in the process of host bacteria adsorption and were thought to be the key factor that determined the phage specificity [20]. A large number of regained-infectivity phage mutants showed mutations in phage tail proteins [48], while no study had reported the synergism mutation of more than one phage tail proteins to be essential for the phage regaining infectivity. In this study, the synergism mutation of nucleotides from three different phage tail proteins, including the baseplate protein (Gp44), the tail endopeptidase protein (Gp46) and the distal tail protein (Gp47), were found to be responsible for the regained infectivity of the phage. The baseplate protein, the tail endopeptidase protein and the distal protein had all been reported to be phage receptor-binding proteins during phage infection [34,48]. All three proteins were located near the baseplate region of the phage particle and were associated with the formation of a phage baseplate substructure. Genome sequencing revealed that the mutations of these nucleotides were also found in reads archived by genome sequencing of the wild-type phage. However, the wild type phage can’t infect the mutant bacterial strains. We inferred that the synergism mutation of these three amino acid residues changed the baseplate structure of the phage and empowered the mutant phage with the ability to bind to a new binding receptor on the phage-resistant bacterial mutants or with a higher ability to bind to the potential binding receptor of the mutated bacterial flagellum.

Although the mutant phage regained its ability to infect the phage-resistant bacterial mutants, its infectivity and stability were significantly decreased. Bioinformatic analysis of the mutant sites in mutant phage isolates revealed that the mutations were located on the protein surface region or could change the secondary structure of these phage baseplate-associated proteins, which were located at the tip of the phage tail and interacted with the receptor at the host surface during the infection process. The mutations of these proteins might change the interaction between themselves or between the other baseplate-associated proteins and reduce the stability of the protein interactions. The reduced protein interaction would lead to the decrease of the stability of phage virion particles and subsequently cause the instability of the phage particle. Although this kind of mutation was not suitable for the long-term persistence of the phage, this kind of mutation could provide the phage with the ability to infect the emergent phage-resistant host mutants and avoid the deracination of the phage. After they had infected into the host cell, the high mutant frequency of the phage could generate new mutations that provided the evolved phage with both a high stability and infectivity to phage-resistant host bacterium. Like a gecko, by using this method of “dying to survive”, the phages perpetuate the continuation of their species in emergent situations and gain new evolution opportunities that could realize the long-term persistence of their genetic information.

In summary, this study figures out the possible resistant mechanism and regains the infectivity mechanism of the bacterium and the phage during the co-evolution process. On one hand, as a widely used recipient strain for constructing genetic engineering strain, BMB171 is a high possibility to be hazarded by phages. The phage-resistant bacterial mutants isolated in this study could be used in the construction of phage-resistant genetic engineering strains for producing biological pesticides. On the other hand, phage therapy is thought as a promising approach for controlling the pathogenic bacteria, while the generation of phage resistance during the use of phage might cause the failure of phage therapy. The understanding of the mechanism of regaining infectivity of phages could provide guidance for constructing genetic engineering phages with infectivity to phage-resistant bacteria.

## Supplementary Materials

The following are available online at http://www.mdpi.com/1999-4915/11/2/118/s1, Table S1: Genome information of bacterial mutants. Table S2: Single nucleotide polymorphism (SNP) analysis of the phage-resistant bacterial mutants. Table S3: Genome information of bacterial mutants. Figure S1: Lytic activity analysis of endolysin PlyBMB encoded by vB_BthS_BMBphi to BMB171 and phage-resistant mutants.
