# Supplementary material for: Phage Reduce Stability for Regaining Infectivity during Antagonistic Coevolution with Host Bacterium
Source: Viruses. 2019 Jan 29;11(2):118. doi: 10.3390/v11020118 (PMC6410104; doi:10.3390/v11020118)
Supplement: Supplementary file 1 [file viruses-11-00118-s001.zip › Supplementary files/Supplementary file 1.docx]

Article

**Phage reduce stability for regaining infectivity during antagonistic coevolution with host bacterium**

Yihui Yuan^1,2^, Qin Peng^3^, Shaowen Zhang^1^, Tingting Liu^1^, Shuo Yang^1^, Qiuhan Yu^1^, Yan Wu^2^, Meiying Gao^2,*^

^1^ State Key Laboratory of Marine Resource Utilization in South China Sea, Hainan University, Haikou 570228, P. R. China.

^2^ Wuhan Institute of Virology, Chinese Academy of Sciences, Wuhan 430071, P. R. China

^3^ Ministry of Education Key Laboratory for Ecology of Tropical Islands, College of Life Sciences, Hainan Normal University, Haikou 571158, P. R. China

**^*^** Correspondence: Meiying Gao, Wuhan Institute of Virology, Chinese Academy of Sciences, Wuhan 430071, P. R. China. Tel +86- 027-87199355, fax +86- 027-87199355, Email: [mygao@wh.iov.cn](mailto:mygao@wh.iov.cn)

Table S1 Genome information of bacterial mutants.

| **Strain** | **Total Reads** | **Total Base (nt0** | **Clean Reads** | **Clean Base （nt）** | **Coverage** | **Q20 (%)** | SRA Accession |
| --- | --- | --- | --- | --- | --- | --- | --- |
| PRB-1 | 15,021,352 | 1,877,669,000 | 14,487,730 | 1,664,252,181 | 294.89 | 92.34 | SRX5189441 |
| PRB-4 | 11,538,482 | 1,442,310,250 | 11,118,192 | 1,275,926,964 | 226.01 | 91.89 | SRX5189442 |
| PRB-5 | 14,830,532 | 1,853,816,500 | 14,300,200 | 1,639,213,714 | 290.60 | 91.38 | SRX5189443 |
| PRB-8 | 12,988,810 | 1,577,942,347 | 12,346,058 | 1,415,972,471 | 251.05 | 92.91 | SRX5189444 |

Q20: Q20=bases of Q>=20 / all bases of sequencing

Table S3 Genome information of bacterial mutants.

| **Strain** | **Total Reads** | **Total Base (nt)** | **Clean Reads** | **Clean Base**  **（nt）** | **Coverage** |  | **Q20 (%)** | SRA Accession |
| --- | --- | --- | --- | --- | --- | --- | --- | --- |
| BMBphi | 11,814,336 | 1,402,673,755 | 10,081,922 | 1,158,200,876 | 23503.88 |  | 93.15 | SRX5189751 |
| RIP-1 | 13,418,606 | 1,677,325,750 | 12,888,764 | 1,477,437,995 | 29982.30 |  | 91.37 | SRX5189752 |
| RIP-2 | 9,209,924 | 1,109,664,499 | 8,177,812 | 941,526,712 | 19106.81 |  | 92.26 | SRX5189753 |
| RIP-4 | 11,018,888 | 1,377,361,000 | 10,594,962 | 1,213,847,625 | 24633.14 |  | 91.32 | SRX5189754 |

Q20: Q20=bases of Q>=20 / all bases of sequencing


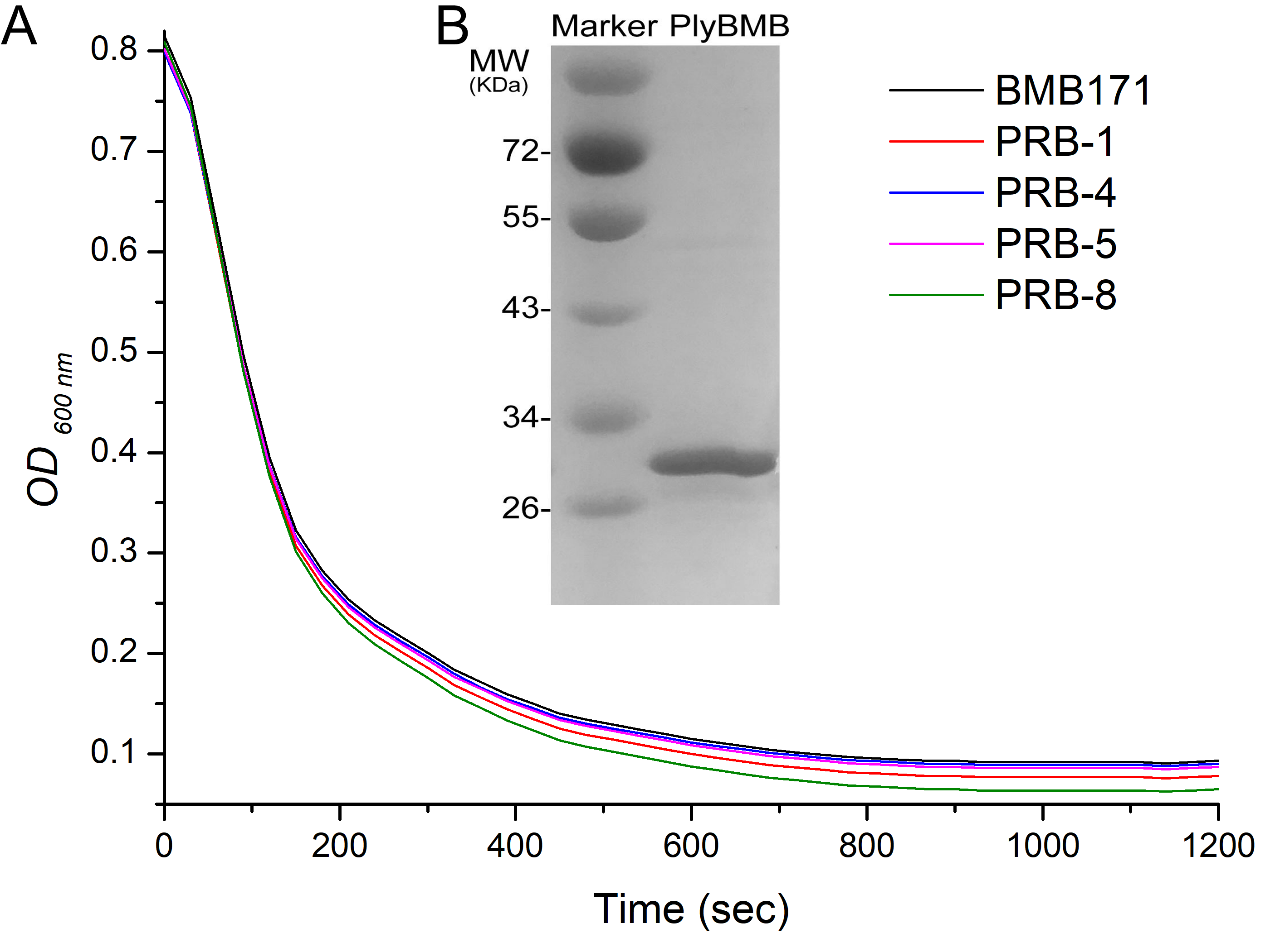


Figure S1 Lytic activity analysis of endolysin PlyBMB encoded by vB_BthS-BMBphi to BMB171 and phage resistant mutants.
